# COVID-19 and blood group-related antigens: can natural anti-carbohydrate antibodies provide innate protection from symptomatic SARS-CoV-2 infection?

**DOI:** 10.3389/fmed.2025.1554785

**Published:** 2025-04-30

**Authors:** Tasnuva Ahmed, Adrien Breiman, Marjahan Akhtar, Golap Babu, Nasrin Pervin, Md Golam Firoj, Afroza Akter, Firdausi Qadri, Fahima Chowdhury, Taufiqur Rahman Bhuiyan, Jacques Le Pendu, Nathalie Ruvoën-Clouet

**Affiliations:** ^1^Infectious Diseases Division, International Centre for Diarrhoeal Disease Research, Dhaka, Bangladesh; ^2^Nantes Université, Univ Angers, INSERM, CNRS, Immunology and New Concepts in ImmunoTherapy, INCIT, UMR 1302, Nantes, France; ^3^CHU de Nantes, Nantes, France; ^4^Oniris-Vet AgroBio Nantes, Ecole nationale, Nantes, France

**Keywords:** Lewis status, secretor status, natural anti-carbohydrate antibodies, COVID-19, Bangladesh, SARS-CoV-2, HBGA, blood group

## Abstract

**Background:**

Severe acute respiratory syndrome coronavirus 2 (SARS-CoV-2) primarily targets respiratory mucosa, causing coronavirus disease 2019 (COVID-19). Susceptibility and severity of COVID-19 may be influenced by predisposing factors including blood groups. In this study, we investigated whether natural anti-carbohydrate antibodies provide innate protection against SARS-CoV-2 and influence disease severity.

**Methodology:**

We used samples (plasma and saliva) from a longitudinal cohort study in Bangladesh that enrolled 100 COVID-19 symptomatic and asymptomatic patients. We also enrolled 21 and 38 healthy controls during the pandemic period and pre-pandemic period, respectively. We phenotype ABO blood grouping from blood and determined Lewis and secretor status (H antigen) from the saliva samples. We quantified natural anti-carbohydrate antibodies (anti-A, anti-B, anti-Tn-Mono and anti-αGal IgG, IgA, and IgM) from plasma collected at enrollment. We also explored the trend of natural anti-carbohydrate antibodies until 3 months of convalescence period among the COVID-19 patients (day 14 and day 90 from enrollment). Antibody quantification and ABH/Lewis phenotyping were performed using enzyme-linked immunosorbent assay (ELISA).

**Results:**

We included 99 COVID-19 patients and 59 healthy controls assessing the differences of natural antibody titer during enrollment, while 95 patients were analyzed exploring Lewis and secretor status with natural antibody titer and disease status. We did not find significant difference in the distribution for neither ABO blood groups nor non-secretors and Lewis-negative individuals among asymptomatic or symptomatic patients and healthy controls. Nonetheless, we observed lower anti-A antibody titers among symptomatic patients compared to healthy controls. We also identified slight differences in antibody titers linked to age and gender. Anti-A and anti-B antibodies among asymptomatic patients had a higher trend up to 3 months from infection compared to symptomatic patients.

**Conclusion:**

Higher natural anti-A and anti-B antibody titers may offer protection against symptomatic COVID-19 infections. Gender and blood group differences indicate potential innate immune factors influencing disease severity, but larger studies are needed to confirm these findings.

## 1 Background

Severe acute respiratory syndrome coronavirus 2 (SARS-CoV-2) targets the respiratory mucosa, and it can infect and replicate in the gastric and intestinal epithelium, causing coronavirus disease 2019 (COVID-19). COVID-19 is a complex and multifactorial disease, where inherited predispositions with comorbidities and risk factors are likely to influence the severity of the disease (1, 2). Most studies have found that blood group O individuals were protected to some extent against SARS-CoV-2 infection while A and/or B blood group individuals were more susceptible to infection (3–6). Some studies also reported some protection of the O individuals against severe disease. Whereas meta-analyses provided evidence that there may be a link between ABO and susceptibility to infection, the link between ABO and severity of the disease appears poorly reliable (7–9). The histo-blood group antigen (HBGA) family, including the ABO blood groups, is expressed on many cell types, particularly epithelial cells of the gastrointestinal tract, upper respiratory tract, and lower genito-urinary tract. Coronaviruses are enveloped viruses whose main envelope protein, the Spike S protein is heavily glycosylated. The sugar coat provides the so-called glycan shield that protects the virus from the adaptive immune response. However, since coronaviruses replicate in cells of the upper respiratory tract, the S protein of virion can express HBGAs, depending on the patient’s genetic polymorphism for these antigens (10, 11).

Individuals who possess the active α1, 2-fucosyltransferase enzyme (FUT2) are known as secretor (Se), while mutations on *FUT2* gene lead to lack or decreased α1,2-fucosyltransferase activity. Therefore, absence of the *α*1, 2 fucosylated antigens in mucosal tissues and secretions (e.g., saliva) results in individual phenotype as non-secretor (se) which represents approximately 20% of Caucasian and African populations (12–15). Similarly, the *FUT3* gene codes an α 1, 3 or 1, 4 fucosyltransferase which can generate Lewis antigens by modifying precursor oligosaccharides (type 1)/H-type 1 or precursor oligosaccharides (type 2)/H-type 2 antigens to form Le^a^ /Le^b^ and Le^x^/Le^y^ antigens, respectively, thus making an individual Lewis-positive (Le+). Lewis negative (le-) individuals lack Le^a^ and Le^b^ regardless secretor status (16). The European population have approximately 4–11% Lewis negative (Le a- b-) and non-secretors (se), whereas approximately 29 and 11% of individuals contribute to African and Asian populations, respectively (15, 17).

Earlier study of SARS-CoV outbreak in Hong Kong during the 2003 outbreak showed that blood group O individuals have low risk of being infected by SARS-CoV-1 compared to non-O blood group individuals (18). The anti-A antibodies were observed to have the ability to inhibit the interaction between SARS-CoV spike protein, produced in cells expressing the A antigen, and its cellular receptor ACE2 (19). It is thus conceivable that this association can be attributed to protection exerted by anti-blood group antibodies and not the blood group antigens (19, 20). The expression of ABH antigens in epithelial cells where SARS-CoV replicates is also controlled by polymorphisms of the *FUT2* gene. Thus, individuals with two *FUT2* null alleles, the so-called non-secretors, are unable to synthesize H antigen and hence A or B antigens in these cells (19).

Since SARS-CoV-2 replicates primarily in epithelial cells of the upper respiratory tract epithelial cells that express these carbohydrate antigens and also use ACE2 as a receptor, our working hypothesis was to verify that the presence of natural anti-carbohydrate antibodies, including anti-blood group A and B antibodies, could confer a certain level of innate protection against infection by SARS-CoV-2 and can explain the association between ABO phenotype and the severity of infection by SARS-Cov2.

## 2 Methods

### 2.1 Study participants

This is a prospective cohort study as mentioned previously (1). In brief, we enrolled 100 COVID-19 patients between November 2020 and February 2021 in Dhaka, Bangladesh. All patients aged 18 years and above were confirmed SARS-CoV-2 reverse transcription polymerase chain reaction (RT-PCR) positive for the first time prior to or during enrollment. We used WHO guideline of COVID-19 (clinical symptoms and oxygen saturation) for determining severity of the patients which were collected from the hospital records on admission or the patient’s condition during enrollment (21) and categorized them into asymptomatic, mild, moderate, and severe (25 patients per severity group). Patients who gave confirmed history of previous SARS-CoV-2 infection with RT-PCR positive results were excluded. Thirty-one age- and sex-matched healthy controls (pandemic controls) were enrolled at the same time period who were judged healthy by medical personnel, had no history of COVID-19, were RT-PCR negative for SARS-CoV-2 during enrollment, and had no clinical signs and symptoms of COVID-19 in the 2 weeks prior to enrollment. Blood specimens from COVID-19 patients for measuring antibody titer were collected prospectively on day 1(enrollment), day 14, and day 90. Saliva specimens were collected on day 1(enrollment), day 7, day 14, and day 28. However, in this analysis, we used saliva specimen only from any one of the day points for Lewis phenotyping and secretory/non-secretory characteristics. As for healthy controls, all samples were collected only during enrollment. ABO blood grouping was done from blood specimen during enrollment by agglutination method. Additional available stored plasma and saliva samples from 38 participants of pre-pandemic period were included in this analysis as a representative of the local population in terms of the distribution of HBGA phenotypes (Lewis and secretor status).

### 2.2 Quantification of natural antibodies in plasma

The circulating anti-carbohydrate antibodies were assessed with the enzyme-linked immunosorbent assay (ELISA) from stored plasma specimen of patients and healthy controls. ELISA plates (F96 Maxisorp, Nunc, Thermo Fisher Scientific, Roskilde, Denmark) were coated with synthetic sugars (from Glyco NZ, Auckland New Zealand): A trisaccharide or A-Tri (GalNAc*α*1,3-(Fucα1,2)-Galß-PAA), B trisaccharide or B-Tri (Galα1,3-(Fucα1,2)-Galß-PAA), Tn monosaccharide or Tn-Mono (GalNAcα-PAA), or α-Gal trisaccharide (Galα1,3-Galβ1,4-GlcNAcβ-PAA) at 10 μg/mL in 0.1 M carbonate buffer pH 9.0 at 4°C overnight. The plates were washed six times in 1X phosphate buffer saline with 0.05% Tween 20 (PBS-0.05%(T), unbound sites were blocked with PBS 5% bovine serum albumin (BSA) for 2 h at 37°C. After six additional washes with PBS-T, plasma samples from COVID-19 patients or controls were added to the plate at a 1:100 dilution in PBS-1% BSA (duplicate wells for coated wells and single non-coated well) for 4 h at 4°C. Optimal dilutions had been chosen based on preliminary analyses performed using plasma samples from healthy blood donors and COVID-19 patients mentioned earlier (20). The plates were then washed six times with PBS-T, and biotinylated Goat anti-human Fcγ (Jackson ImmunoResearch Laboratories Inc., Ely, United Kingdom), biotinylated Goat anti-human IgA (Novus), and biotinylated-Goat anti-human IgM (Novus) were added at a 1/10000, 1/5000, and 1/5000 dilutions, respectively, in PBS-1% BSA for 1 h at 37°C. After another six washes with PBS-T, secondary conjugate Avidin-HRP (vector laboratories) was added to the plates at 1:3000 dilution in PBS-1% BSA and incubated for 1 h at 37°C. Finally, after five last washes with PBS-T and one with plain PBS, revelation was performed with 50 μL/well of 3,3′,5,5′-tetramethylbenzidine (Sigma Aldrich, St Louis, MO), and the reaction was stopped with 50 μL/well of 1 M phosphorous acid after 3 min. Optical densities were read twice at 450 nm with an EON BioTek spectrophotometer. The final value was obtained after subtracting the background value (non-coated wells) from the average value of the coated wells.

### 2.3 Secretor and Lewis phenotyping by ELISA on saliva

ABH and Lewis phenotyping was done by enzyme-linked immunosorbent assay (ELISA) on saliva. Saliva specimens were boiled for 10 min and briefly centrifuged, and the supernatants were diluted in carbonate–bicarbonate buffer solution at 1:1000 along with control positive saliva. ELISA plates (NUNC 96F Maxisorp; Thermo Fisher Scientific, Roskilde, Denmark) were coated with the diluted mucins, sealed with parafilm, and incubated at 4°C overnight. On the following day, plates were first washed with PBS-0.05%(T) for three times and then saturated in PBS 5% milk to block the non-specific sites and incubated for 1 h at 37°C in humid atmosphere. Afterward, the plates were washed again like before and 100 μL per well of primary antibodies anti-Lewis a (7Le, Thermo Fisher Scientific) and anti-Lewis b (2-25Le, Thermo Fisher Scientific) diluted at 1:500 in 5% PBS milk were introduced accordingly. After three washes with PBS-0.05%(T), plates were coated with secondary reagents anti-mouse-HRP (Uptima up446330, Interchim, Montluçon, France) at 1:1000 in 5% PBS milk and incubated for 1 h at 37°C. To determine the secretor status, 100 μL/well of biotinylated UEA-I lectin anti-H type 2 (Vector Laboratories, Newark, CA) were added to the corresponding wells. The lectin UEA-I was diluted at 1:500 saturated in PBS 5% milk. The plates were incubated for 1 h at 37°C like before and then coated with secondary reagents streptavidin-HRP (Vector Laboratories) at 1:3000 dilution in 5% PBS milk after three washes like before.

Finally, after three washes with PBS-0.05%(T) and two with PBS, reaction was initiated with 50 μL of 3,3′,5,5′-tetramethylbenzidine (TMB) per well (BD OptEIA, BD Biosciences), incubated for 5–7 min at room temperature, and afterward stopped by loading 50 μL of 1 M phosphoric acid. Optical density (OD) of each plate was read at 450 nm by EON BioTek, and values equivalent to twice the background were considered positive. Individuals with a positive response to Ulex were considered to be secretors, and individuals with a positive response to anti Lewis a and/or or anti Lewis b were considered to be Lewis positive.

### 2.4 Statistical analysis

The mild, moderate, and severe COVID-19 patients from the original cohort were grouped into symptomatic COVID-19 patients. Demographic information, blood group (A, B, AB, and O), and clinical characteristics of the participants were stratified by health status (healthy control, asymptomatic, and symptomatic). Antibody titer was measured as geometric mean (GM) with 95% confidence interval (CI). Continuous variables were described as mean with 95% confidence interval (CI) or median with inter-quartile range (IQR) and frequency with percentage for categorical data. To identify the significant difference, *t*-test was used to compare the mean among the different groups, while for median and percentage, non-parametric Kruskal–Wallis rank-sum test or chi-square test was used, respectively. Antibody titers were measured as geometric mean (GM) with 95% CI. To assess the difference in antibody response between the groups, we conducted linear regression analysis on log-transformed antibody titers adjusted for age. We included age as a covariate to adjust for its potential confounding effect and an interaction term between age and symptom status to evaluate whether age modifies the relationship between symptom status and antibody titers. The significance of the coefficients of the models was tested using *t*-test that represents the difference in the log-transformed means between the groups adjusted for age. Since patients were enrolled at different time points from disease onset, we analyzed the trend of antibody titers over the convalescent period by categorizing the intervals from the date of disease onset to study follow-up dates, up to study day 90. Box plots and line plots with scattered points were created to see the distribution of clinical laboratory values in different groups. All analyses were done using GraphPad Prism version 6.0 and R statistical software version 4.2.2 (“ggplot2” and “ggpubr” packages for the scatter and boxplot diagram and “dplyr” package for data).

## 3 Results

### 3.1 Demographic

**Figure 1:**
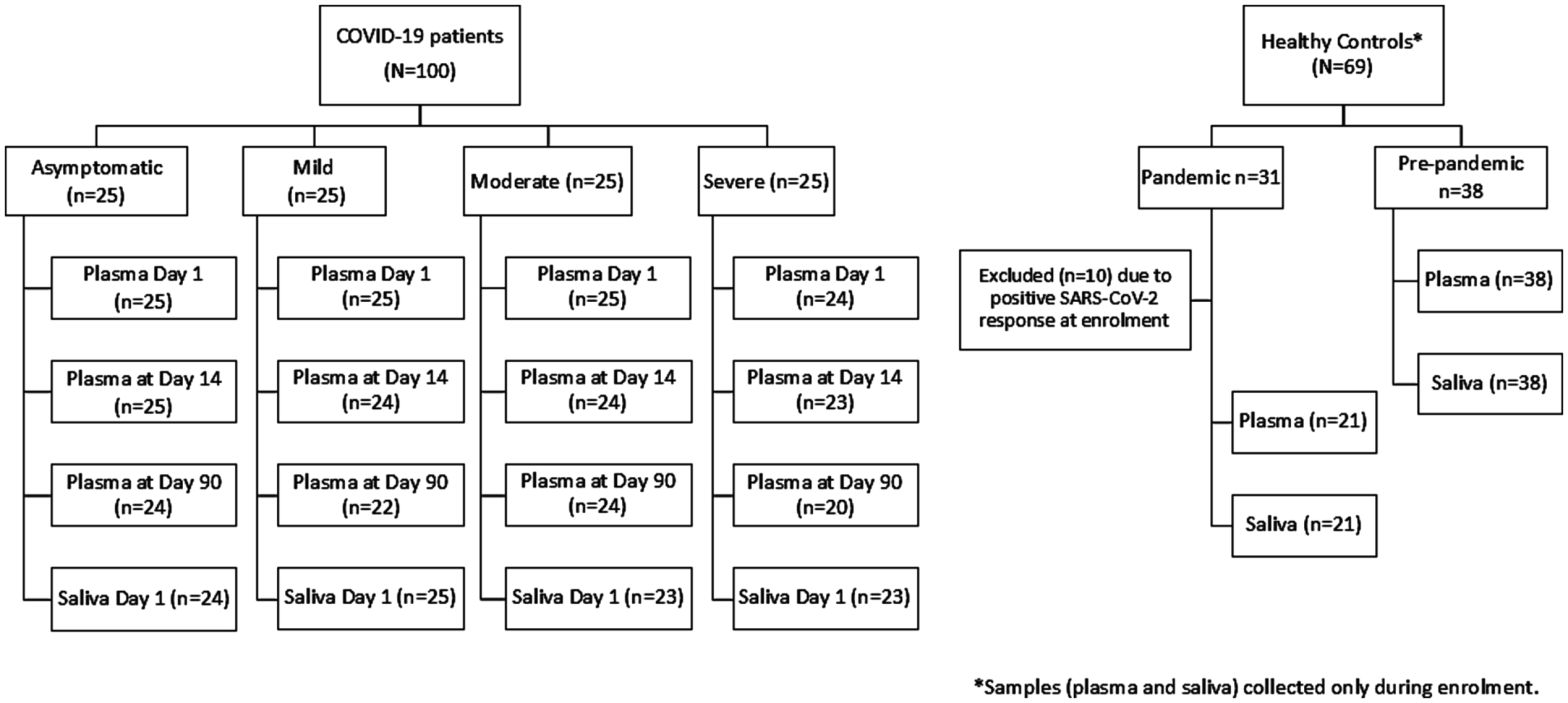
Participant flow diagram for blood and saliva sample collection.

A total of 99 COVID-19 patients (asymptomatic, *n* = 25; mild, *n* = 25; moderate, *n* = 25; and severe, *n* = 24) and 59 healthy controls were included in the analysis who had plasma during enrollment (Figure 1). From the pandemic healthy control participants, 10 participants were excluded from the final analysis due to SARS-CoV-2 RBD-specific antibody response.

The overall median age of the COVID-19 patients in this cohort was 46 years (IQR: 35, 57.5). Asymptomatic infection was observed among the younger patients [median age 35 years (IQR: 31, 44)], while older patients suffered from symptomatic SARS-CoV-2 infection [median age 50 years (IQR: 38, 62)] and the median age of healthy control (both pandemic and pre-pandemic) was 33 (IQR: 28.5, 41) (Table 1). Male patients suffered mostly from symptomatic infection (69%), while majority of the female patients (64%) had asymptomatic infection. Distribution of individuals by blood group was similar between the patient and healthy control populations.

**Table 1:** Demographic and distribution of histo-blood group antigens among the COVID-19 patients and controls.

Parameters	All patients *n* = 99	Asymptomatic *n* = 25	Symptomatic^a^ *n* = 74	Healthy controls and pre-pandemic^b^ *n* = 59	*p*-value^c^
Sex					
Female	39 (39.4%)	16 (64.0%)	23 (31.1%)	27 (45.8%)	**0.011**
Male	60 (60.6%)	9 (36.0%)	51 (68.9%)	32 (54.2%)	
Age Median (IQR)	46 (35, 57.5)	35 (31, 44)	50 (38, 62)	33 (28.5, 41)	**<0.001** ^**d**^
Blood group					
A Blood group	25 (25.3%)	8 (32.0%)	17 (23.0%)	17 (28.8%)	0.791
AB Blood group	9 (9.1%)	2 (8.0%)	7 (9.5%)	3 (5.1%)	
B Blood group	35 (35.3%)	7 (28.0%)	28 (37.8%)	17 (28.8%)	
O Blood group	30 (30.3%)	8 (32.0%)	22 (29.7%)	22 (37.3%)	
Duration between disease onset and enrollment, Median days (IQR)	10 (8, 12)	10 (2, 16)	10 (8, 12)	–	0.680^d^

Data presented as *n* (%).

^a^Symptomatic patient: mild: n = 25, moderate: n = 25, severe: n = 24.

^b^Pandemic healthy controls: n = 21; pre-pandemic healthy controls: n = 38.

^c^Pearson’s chi-squared test for association.

^d^Non-parametric Kruskal–Wallis rank-sum test.

Bold values indicate significant difference between asymptomatic, symptomatic and healthy controls.

In this cohort, we phenotyped Lewis and secretor status from 95 COVID-19 patients who had sufficient saliva samples stored. Among the healthy controls, 30.5% were non-secretors, whereas 25% asymptomatic patients and 40.8% symptomatic patients were non-secretors. Lewis negative individuals (Lewis a- b-) among the control groups were 11.9%, while asymptomatic and symptomatic patients consisted of 20.8 and 16.9% individuals (Table 2). However, no significant difference was found between the control group and the patient group for the secretor and Lewis characteristics.

**Table 2:** Distribution of Lewis and secretor status by asymptomatic, symptomatic, and healthy control group.

Label	Asymptomatic, *n* = 24	Symptomatic, *n* = 71	Control, *n* = 59*	*p*-value^$^
Lewis status				
Lewis positive *n* = 130	19 (79.17%)	59 (83.10%)	52 (88.14%)	0.544
Lewis negative *n* = 24	5 (20.83%)	12 (16.90%)	7 (11.86%)	
Secretor status				
Secretor, *n* = 101	18 (75%)	42 (59.15%)	41 (69.49%)	0.267
Non-secretor, *n* = 53	6 (25%)	29 (40.85%)	18 (30.51%)	

^$^Chi-square test.

^*^Pandemic healthy control, *n* = 21; pre-pandemic healthy control, *n* = 38.

### 3.2 Age stratified comparison of natural antibody titer

Given the age differences among healthy controls, asymptomatic, and symptomatic COVID-19 patients, we stratified participants into two groups: <45 and ≥45 years. Anti-B IgG and IgA titers were significantly higher in asymptomatic patients <45 years compared to both symptomatic patients and healthy controls in the same age group (Supplementary Figure 1B). Younger healthy controls (<45 years) had higher anti-A IgM titers than older controls (Supplementary Figure 1I). Asymptomatic patients <45 years also showed significantly elevated anti-αGal IgG, IgA, and IgM titers compared to healthy controls (Supplementary Figures 1C,G,K). No significant differences were observed in Tn-Mono antibody titers across groups (Supplementary Figures 1D,H,L).

The regression analysis on antibody titer and adjusted age (Supplementary Table 1) indicates that age and symptomatic status were not significantly associated with IgA antibody levels or with IgG antibody levels against anti-A, anti-Tn, and anti-*α* Gal. However, for anti-B IgG, symptomatic status was significantly associated with lower titers (*p* = 0.002), while age alone was not a significant predictor. In addition, among anti-Tn-Mono IgM titer, age showed a modest but significant negative association (*p* = 0.030). The interaction term between age and symptomatic status was significant for both anti-B IgG (*p* = 0.018) and anti-Tn-Mono IgM (*p* = 0.047), suggesting that the effect of age on these antibody levels may differ depending on symptom status.

### 3.3 Natural antibody titers among different blood groups, COVID-19 patients, and healthy controls

**Figure 2:**
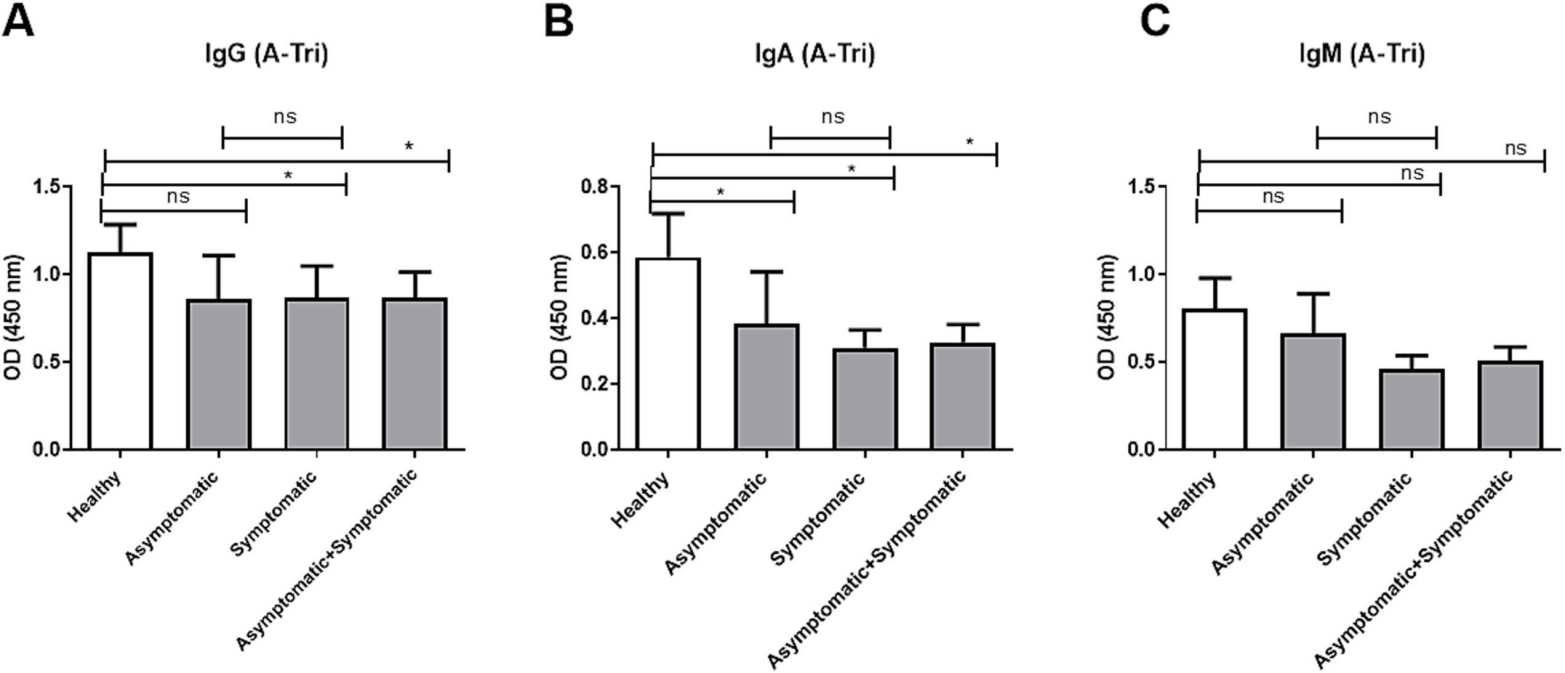
Anti-A antibody titer in O and B blood group patients and controls. Comparison of anti-A antibody (A-Tri) titer between healthy control (*n* = 39), asymptomatic patients (*n* = 15), symptomatic patients (*n* = 50), and combined (asymptomatic+ symptomatic) patients (*n* = 65) of individuals with O and B blood groups. **(A)** Anti-A IgG antibody, **(B)** anti-A IgA antibody, and **(C)** anti-A IgM antibody. *** and ** denote *p* < 0.005.

Since anti-A antibody is present among blood groups O and B, we compared the age-adjusted IgG, IgA, and IgM titers between healthy controls, asymptomatic, and symptomatic COVID-19 patients from O and B blood group individuals only (Figure 2). Anti-A IgG and IgA antibody titers (Figures 2A,B) were significantly lower in both symptomatic and combined (asymptomatic + symptomatic) COVID-19 patient groups compared to healthy controls, while no significant difference was observed between asymptomatic and symptomatic patients. In contrast, anti-A IgM titers did not differ significantly among any of the groups (Figure 2C).

**Figure 3:**
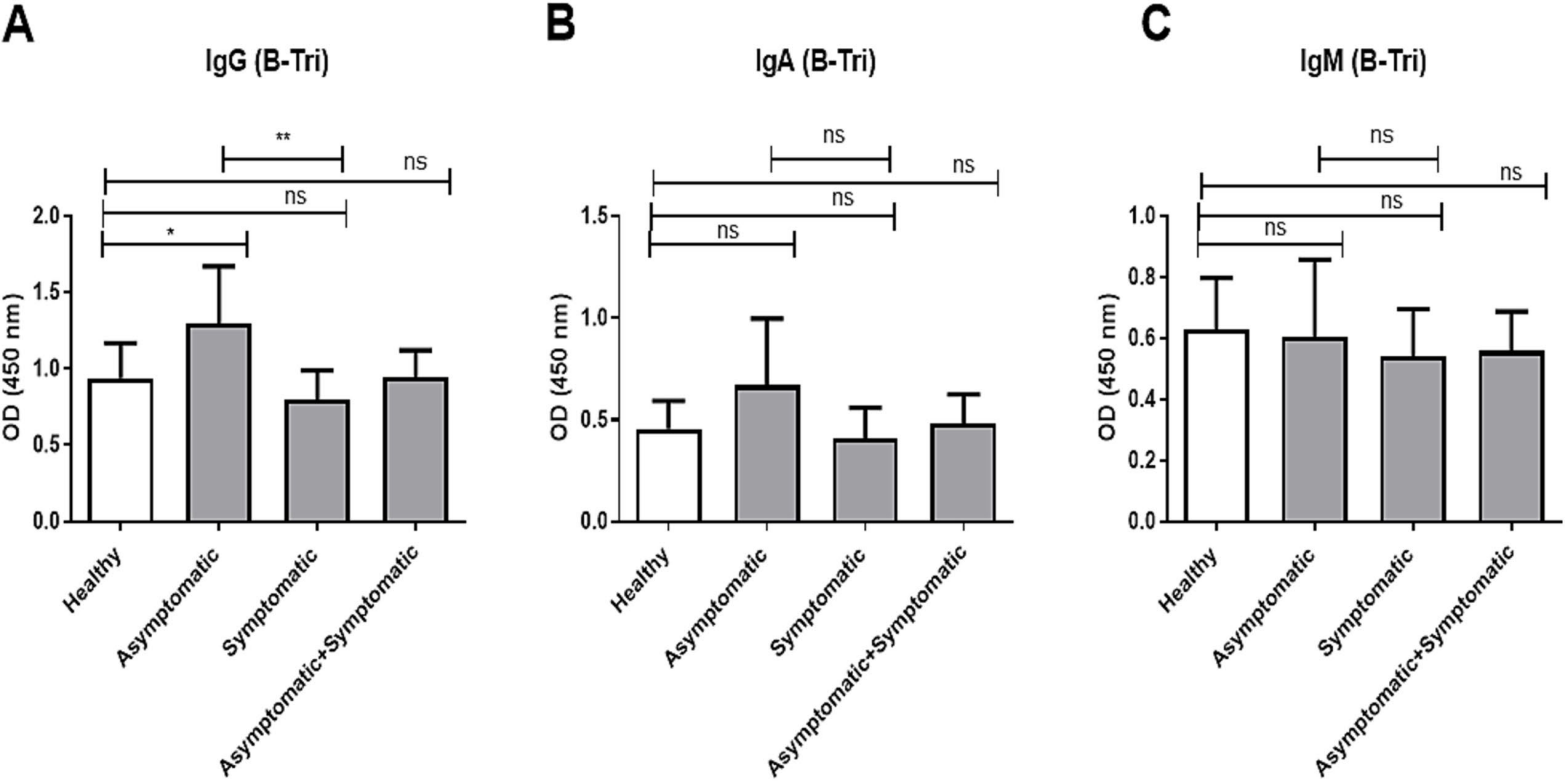
Anti-B antibody titer in O and A blood group patients and controls. Comparison of anti-B antibody (B-Tri) titer between healthy control (*n* = 39), asymptomatic patients (*n* = 16), symptomatic patients (*n* = 39), and combined (asymptomatic+ symptomatic) patients (*n* = 56) of individuals with O and A blood groups. **(A)** Anti-A IgG antibody, **(B)** anti-A IgA antibody, and **(C)** anti-A IgM antibody. *** and ** denote *p* < 0.005.

Similarly, we compared age-adjusted anti-B IgG, IgA, and IgM antibody titers among O and A blood group individuals, across healthy controls, asymptomatic, and symptomatic COVID-19 patients (Figure 3). For anti-B IgG (Figure 3A), asymptomatic individuals had significantly higher titers compared to healthy controls (*p* < 0.05) and significantly higher than symptomatic patients (*p* < 0.01), while no significant difference was observed between symptomatic patients and healthy controls. In contrast, no significant differences were observed in anti-B IgA (Figure 3B) or IgM (Figure 3C) titers among any of the groups.

We further explored the anti-Tn and anti-*α* Gal age-adjusted titers. Since the Tn-Mono antigen (*α*GalNAc) and the αGal antigen (Galα1,3-Galß1,4-GlcNAc) are structurally close, respectively, to the A and B antigens, we compared the anti-Tn titers between “non-A” (B + O) versus “A” blood groups (A + AB) and the anti-*α*Gal titers between “non-B” (A + O) versus “B” (B + AB) blood groups, respectively (Supplementary Figure 2). We compared the antibody titer of the respective blood groups within the COVID-19 patients, within the healthy control and between the patients and healthy control groups, respectively. The anti-Tn IgA and IgM antibody titers were significantly higher among the “non-A” blood group than the “A” blood groups (A + AB) within COVID-19 patients (Supplementary Figures 2C,E). Similarly, the anti-α gal antibody titers were significantly higher among the “non-B” blood groups than the “B” blood groups (Supplementary Figures 1B,D,F). Interestingly, the anti-α-Gal antibody titers were found to be significantly higher in COVID-19 non-B blood group patients compared to healthy controls.

Comparing the differences of natural antibody titers among the COVID-19 patients and controls stratified by secretor status, we observed secretor healthy controls had significantly higher anti-A IgG (*p* = 0.006) antibody titer than secretor patients (Table 3). However, no difference was observed in anti-A IgA (*p* = 0.081) and IgM (*p* = 0.663) antibody titer between the secretor patients and controls. No significant differences in anti-B IgG, anti-Tn-Mono, or anti-α Gal antibody titers were observed between healthy controls and patients, regardless of secretor status (Table 3).

**Table 3:** Natural antibody titer according to secretor status by COVID-19 patients and healthy controls.

Factor	Labels	IgA	*p*-value^$^	IgG	*p*-value^$^	IgM	*p*-value^$^
A-Tri (B + O)							
Secretors	COVID-19 Patients (*n* = 39)	0.26 (0.21, 0.33)	0.081	0.62 (0.48, 0.81)	**0.006**	0.41 (0.32, 0.54)	0.663
	Control (*n* = 27)	0.44 (0.33, 0.58)		0.97 (0.80, 1.19)		0.62 (0.45, 0.85)	
Non-secretors	COVID-19 Patients (*n* = 24)	0.26 (0.19, 0.35)	0.144	0.73 (0.51, 1.04)	0.722	0.40 (0.33, 0.49)	0.838
	Control (*n* = 12)	0.49 (0.32, 0.76)		0.92 (0.60, 1.42)		0.50 (0.27, 0.94)	
B-Tri (A + O)							
Secretors	COVID-19 Patients (*n* = 32)	0.34 (0.26, 0.45)	0.639	0.66 (0.51, 0.85)	0.840	0.47 (0.35, 0.63)	0.596
	Control (*n* = 28)	0.33 (0.26, 0.42)		0.68 (0.50, 0.91)		0.45 (0.32, 0.63)	
Non-secretors	COVID-19 Patients (*n* = 23)	0.36 (0.27, 0.48)	0.238	0.88 (0.67, 1.16)	0.319	0.32 (0.23, 0.44)	0.692
	Control (*n* = 11)	0.36 (0.21, 0.62)		0.74 (0.41, 1.31)		0.43 (0.27, 0.68)	
Tn-Mono (A + AB)							
Secretors	COVID-19 Patients (*n* = 23)	0.38 (0.31, 0.46)	0.982	0.64 (0.53, 0.78)	0.623	0.69 (0.53, 0.89)	0.348
	Control (*n* = 14)	0.37 (0.30, 0.46)		0.73 (0.53, 1.00)		0.87 (0.71, 1.08)	
Non-secretors	COVID-19 Patients (*n* = 11)	0.31 (0.24, 0.39)	0.472	0.55 (0.43, 0.70)	0.664	0.63 (0.44, 0.91)	0.448
	Control (*n* = 6)	0.40 (0.26, 0.62)		0.66 (0.35, 1.27)		0.88 (0.64, 1.20)	
α-Gal (B + AB)							
Secretors	COVID-19 Patients (*n* = 30)	0.53 (0.45, 0.62)	0.712	1.27 (1.14, 1.41)	0.159	0.87 (0.74, 1.02)	0.948
	Control (*n* = 13)	0.53 (0.45, 0.63)		1.01 (0.87, 1.17)		0.89 (0.72, 1.12)	
Non-secretors	COVID-19 Patients (*n* = 12)	0.45 (0.35, 0.59)	0.838	1.03 (0.76, 1.39)	0.664	0.67 (0.51, 0.90)	0.689
	Control (*n* = 7)	0.49 (0.37, 0.64)		1.13 (0.90, 1.41)		0.79 (0.65, 0.95)	

^$^t-test for difference adjusted by age. *p* < 0.05 indicates significant difference between the groups.

Data presented as geometric mean (95% CI).

Bold values indicated a significant difference between COVID-19 patients and controls.

### 3.4 Gender-stratified difference of natural antibody titers among COVID-19 patients and healthy controls

**Figure 4:**
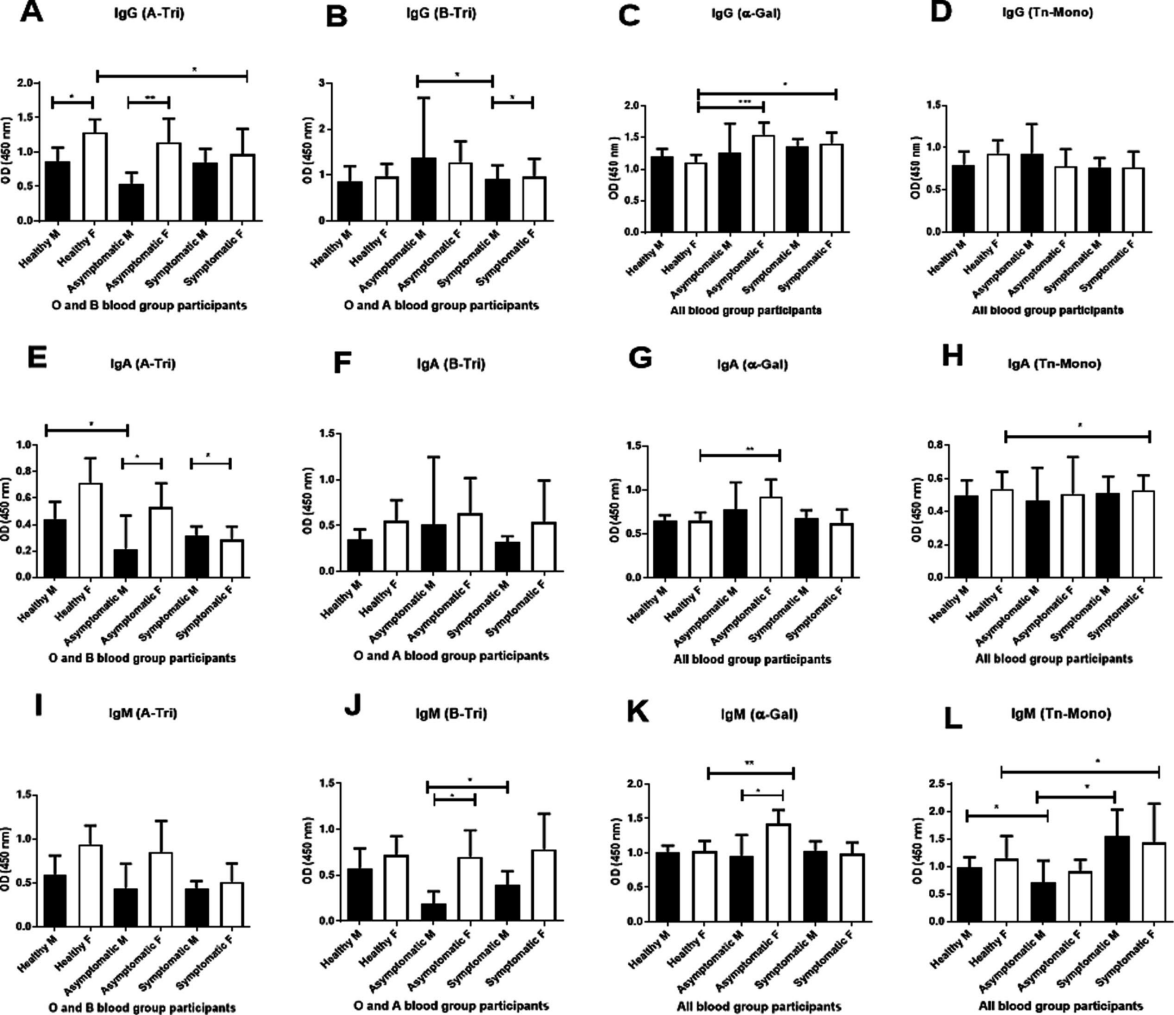
Gender-based analysis of natural antibody. Comparison of natural anti-A (A-Tri), anti-B (B-Tri), anti *α*-Gal, and anti-Tn-Mono (Tn-Mono) IgG **(A–D)**, IgA **(E–H)** and IgM **(I–L)** antibodies between healthy controls, asymptomatic, and symptomatic patients stratified by gender. For anti-A antibody: healthy male (M) *n* = 18, female (F) *n* = 21; asymptomatic male (M) *n* = 6, female (F) *n* = 8; symptomatic male (M) *n* = 33, female (F) *n* = 15. Anti-B IgG antibody (healthy male = 21, female = 18), asymptomatic male (M) *n* = 3, female (F) *n* = 13; symptomatic male (M) *n* = 24, female (F) *n* = 15; Alpha-Gal or Tn-Mono (healthy male = 32, female = 27); asymptomatic male (M) *n* = 8, female (F) *n* = 16; symptomatic male (M) *n* = 49, female (F) *n* = 23. Asterisks *** and ** denote statistical significance, *p* < 0.005.

We further explored the age-adjusted differences in natural antibody titer between male and female COVID-19 patients and controls (Figure 4). Anti-A IgG titers (Figure 4A) were significantly higher in female healthy controls and female asymptomatic patients compared to their male counterparts.

Anti-B IgG titer (Figure 4B) was significantly higher in asymptomatic females than symptomatic males.

Anti-αGal IgG titer (Figure 4C) was elevated in both asymptomatic and symptomatic females than healthy female controls.

For IgA, anti-A IgA titer (Figure 4E) was higher in female healthy controls (O and B blood groups) than in healthy males and symptomatic females. Anti-αGal IgA titers (Figure 4G) were elevated in asymptomatic females than both healthy and symptomatic females. Anti-Tn IgA titers (Figure 4H) were higher in females (pooled) than males. In the IgM isotype, anti-A IgM titer (Figure 4I) was higher in female healthy controls and asymptomatic patients than in male counterparts, and lower in symptomatic females compared to healthy females. Anti-B IgM titer (Figure 4J) was significantly higher in asymptomatic and symptomatic females than in corresponding males. Anti-αGal and anti-Tn IgM titers were significantly higher in asymptomatic females compared to symptomatic and healthy females, and in both asymptomatic and symptomatic females than their male counterparts (Figures 4K,L).

### 3.5 Trend of natural antibody titer over convalescence period among COVID-19 patients

**Figure 5:**
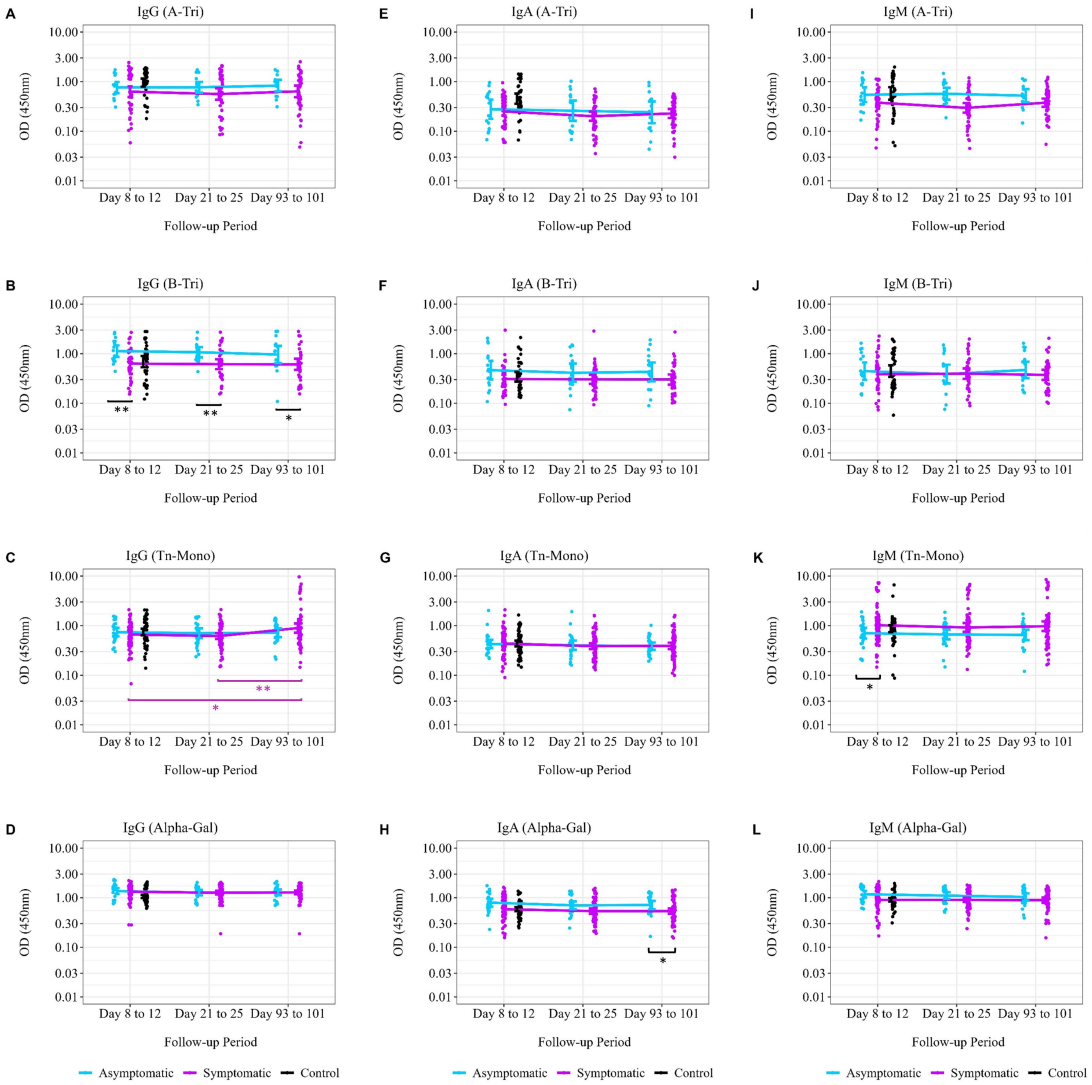
Trend of natural antibodies over 3 months post-SARS-CoV-2 infection. Comparing the trend of natural antibody dynamics over the convalescence period from date of disease onset of exposure. The follow-up period, displayed on the *x*-axis, was calculated from the date of disease onset to each study follow-up time point. **(A)** Anti-A IgG antibody titer (asymptomatic, *n* = 15; symptomatic, *n* = 50; control, *n* = 39), **(B)** anti-B IgG antibody titer (asymptomatic, *n* = 16; symptomatic, *n* = 39; control, *n* = 39), **(C)** anti-Tn-Mono IgG antibody titer (asymptomatic, *n* = 25; symptomatic, *n* = 74; control, *n* = 59), and **(D)** anti-αGal IgG antibody titer (asymptomatic, *n* = 25; symptomatic, *n* = 74; control, *n* = 59). **(E)** Anti-A IgA antibody titer (asymptomatic, *n* = 15; symptomatic, *n* = 50; control, *n* = 39), **(F)** anti-B IgA antibody titer (asymptomatic, *n* = 16; symptomatic, *n* = 39; control, *n* = 39), **(G)** anti-Tn-Mono IgA antibody titer (asymptomatic, *n* = 25; symptomatic, *n* = 74; control, *n* = 59), and **(H)** anti-αGal IgA antibody titer (asymptomatic, *n* = 25; symptomatic, *n* = 74; control, *n* = 59). **(I)** Anti-A IgM antibody titer (asymptomatic, *n* = 15; symptomatic, *n* = 50; control, *n* = 39), **(J)** anti-B IgM antibody titer (asymptomatic, *n* = 16; symptomatic, *n* = 39; control, *n* = 39), **(K)** anti-Tn-Mono IgM antibody titer (asymptomatic, *n* = 25; symptomatic, *n* = 74; control, *n* = 59), and **(L)** anti-αGal IgM antibody titer (asymptomatic, *n* = 25; symptomatic, *n* = 74; control, *n* = 59). The purple asterisks denote geometric mean of antibody titer comparisons between two time points among symptomatic patients, while black asterisks denote difference in geometric mean antibody titer between the asymptomatic and symptomatic patients at a single time point. * denotes *p* = 0.05–0.01; ** denotes *p* = 0.01–0.001; and *** denotes *p* < 0.001.

We further explored the trend of natural antibody titer during the convalescence period from the disease onset or exposure (asymptomatic cases) stratified by symptomatic status and blood group. Anti-A antibody (IgG and IgA) among blood groups B and O individuals remained steady over time, except for anti-A IgM, which showed a significant decrease among symptomatic patients between Days 8 to 12 and Days 21 to 25 (Figure 5I). However, for anti-B IgG antibody among blood group A and O individuals, there were significant differences between symptomatic and asymptomatic patients at Days 8 to 12 (*p* = 0.0038) and Days 21 to 25 (*p* = 0.004) (Figure 5B) from disease onset. The Tn-Mono antibody titer for IgG among the symptomatic patients with all blood groups significantly increased after 3 months (*p* = 0.01 between Days 8 to 12 and Days 93 to 101; *p* = 0.003 between Days 21 to 25 and Days 93 to 101) (Figure 5C). Additionally, anti-Tn-Mono IgM antibody titers were higher in symptomatic patients compared to asymptomatic patients, and this differences persisted throughout the convalescence period (Figure 5K). Although no increase was observed in alpha-gal antibody titer over the 3 months, however, a significant difference in IgA and IgM was observed between the symptomatic and asymptomatic patients (Figures 5H,L).

## 4 Discussion and conclusion

This is the very first study in South Asia looking into the interference of host natural antibody in relation to blood group status with symptomatic of SARS-CoV-2 infection. The current study was part of an exploratory analysis on the relation of natural anti-carbohydrate antibodies, Lewis phenotype, and secretor status with COVID-19 disease severity. The demographic characteristics in our cohort were described previously in our first study (1) having younger patients of median age 35 years old suffering from asymptomatic SARS-CoV-2 infection while elder patients of median age 50 years old suffering mostly from symptomatic infection. As discussed earlier, male patients suffer more from severe disease than female patients and majority of the asymptomatic infection was presented by female patients (1). Thus, from this analysis, we have further investigated the host factors which may support the reason behind this trend of SARS-CoV-2 infection.

In our cohort, we found 38% of the COVID-19 patients with B blood group suffered from symptomatic SARS-CoV-2 infection while O blood group patients represented 33% of asymptomatic infections and 37% of healthy controls. The association between blood group type and susceptibility to symptomatic COVID-19 has been a topic of research. Studies have suggested that individuals with O blood type may have a lower risk of developing severe symptoms or being infected with SARS-CoV-2 (2, 22). However, in our cohort, we did not find any difference in the proportion of A, B, AB, and O blood group distribution among the asymptomatic, symptomatic, and healthy controls which was debatable with the results obtained in other studies where blood group A was predominant among the COVID-19 patients (4, 23). It has been discussed elsewhere that O blood group individuals naturally possess both anti-A and anti-B antibodies which may provide protection against SARS-CoV-2 by preventing the binding of SARS-CoV-2 to its receptor thereby inhibiting the virus entry into the targeted human cells (24, 25). As discussed above, the presence of the HBGAs in the epithelial cells has previously been implicated in the genetic susceptibility to several infectious diseases, including viral diseases (24). Previously, our group have shown that anti-A could block the binding of SARS-CoV spike protein produced in a A antigen positive cell to ACE2 (19), while another group have shown that SARS-CoV-2 spike can bind to the A antigen (like other viral proteins such as some norovirus strain VP1 or some rotavirus strain VP8 capsid proteins) (26). These two distinct mechanisms could explain that different blood groups have different susceptibility to SARS-CoV-2 infection. The H-antigen and A/B antigens in respiratory cells may enhance SARS-CoV-2 binding to host receptors, influencing infection susceptibility (2). In this cohort, we have observed approximately 30.5% individuals with inactive *FUT2* enzymes making them non-secretors and 11.86% Lewis negative individuals among the control groups (Table 2) which is similar to other studies in Bangladesh and Asian population (27–29). However, this distribution is higher than most Caucasian population and Chinese population (2, 30–33).

Natural antibodies are part of the innate immune system, which is the first line of defense against infections in the human body (34). Previous studies, including observations from the SARS-CoV 2003 outbreak and other viral infections, suggest that anti-A and anti-B antibodies can inhibit viral entry by blocking interactions between viral proteins and host blood group antigens (19). In our cohort, anti-A and anti-B antibody titers did not differ significantly between healthy controls, asymptomatic, and symptomatic patients when stratified by age (<45 vs. ≥45 years), suggesting that age is not a confounding factor in the observed association between lower natural antibody titers and symptomatic COVID-19 (Supplementary Figure 1). A minor difference in anti-αGal IgG titers observed among healthy controls likely reflects sampling variability due to small group size and should be interpreted cautiously. Larger studies are needed to further confirm that age does not influence natural antibody levels in the context of SARS-CoV-2 infection.

Our data showed that anti-A IgG and IgA titers were significantly lower in symptomatic and combined COVID-19 patient groups compared to healthy controls (Figure 2), while anti-B IgG was significantly lower in symptomatic patients compared to asymptomatic patients but not compared to controls (Figure 3). This finding is consistent with the observation from the study conducted in Belgium that hospitalized patients who had lower anti-A and/ or anti-B IgM antibody titer compared to healthy control were at risk of developing SARS-CoV-2 infection (35). The elevated anti-A and anti-B IgG levels observed in asymptomatic individuals may reflect a protective antibody profile, while IgA and IgM levels remained unchanged between patient groups (24, 35). This is further supported by epidemiological data, including our review of 35 studies, which found that blood group A (who lack anti-A antibodies) was associated with higher infection risk in over 50% of studies, compared to only 20% for blood group B (who lack anti-B) (24). These findings reinforce the hypothesis that anti-A antibodies may provide stronger protection than anti-B, possibly due to the presence of A-like antigens on the viral envelope.

The increased susceptibility of blood group A supports the idea that anti-A antibodies may interfere with viral binding to ACE2, reducing viral entry and disease severity. Collectively, these results highlight the potential role of natural anti-carbohydrate antibodies in modulating COVID-19 susceptibility and clinical outcome. Histo-blood group antigens (HBGAs) are expressed on various epithelial surfaces, including those in the respiratory and digestive tracts, where SARS-CoV-2 is known to replicate and be transmitted. The viral spike protein is heavily glycosylated, and the specific glycan structures it carries depend on the host cell type (24, 35). However, glycans detected on recombinant spike protein differ from those found on viral particles produced by infected individuals’ epithelial cells, making recombinant spike unreliable for inhibition studies involving anti-A or anti-B antibodies (2). Given this discrepancy, we focused on studying and comparing natural antibody titers (anti-A and anti-B) between COVID-19 patients and healthy controls, particularly regarding secretor status.

The secretor status (*FUT2* gene) and Lewis blood group system (*FUT3* gene) influence mucosal antigen expression, impacting SARS-CoV-2 susceptibility and immune response (36). Since secretor individuals express ABO and Lewis antigens on mucosal surfaces, their viral particles may acquire host-derived glycans, potentially altering immune recognition and neutralization. In contrast, non-secretors lack these antigens, which may limit viral binding and reduce disease severity, as observed in gastroenteritis cases such as rotavirus infection making non-secretors less susceptible to rotavirus (37). It is anticipated that infectious viral particles generated by respiratory epithelial cells in individuals of the “secretor” phenotype would bear the H, A, and B antigens (24). Therefore, we compared the anti-A and anti-B antibody titer between the COVID-19 patients and controls stratified by secretor status.

Our findings revealed that secretor healthy controls (B and O groups) had higher anti-A antibody titers compared to COVID-19 patients, suggesting a protective role of these antibodies (Table 3). In addition, Lewis-negative individuals (Le a- b-) have been associated with lower hospitalization rates, further supporting a potential protective effect (2).The significant reduction in IgA and IgM levels in secretor COVID-19 patients may indicate immune modulation tied to prolonged antigen exposure, leading to immune tolerance or antigen–antibody complex formation, which can dampen mucosal immunity (36). Since IgA is important for respiratory defense, lower levels in secretors may impair mucosal protection, increasing viral shedding and disease severity (24). These findings emphasize that host glycan expression plays a key role in shaping SARS-CoV-2 susceptibility, immune response, and disease outcomes although a larger study is required to determine the magnitude of the effect of secretor status and Lewis phenotypes on SARS-CoV-2 infections (2).

We observed a consistent trend of higher anti-A (IgG, IgM, and IgA) and anti-B IgM antibody titers in female healthy controls and asymptomatic patients compared to their male counterparts (Figure 4). Although not statistically significant for all groups, there is a trend of higher ABO antibody titer among the female individuals than male (Figure 4). Similar phenomenon of higher ABO antibody titer among female healthy blood donors than male healthy donors has been observed in a Japanese population (38). This finding further supports the hypothesis that natural antibodies may protect against severity of COVID-19, and hence female suffers milder or asymptomatic infection compared to male individuals (Figure 4) (1, 34, 35).

Comparing the anti-A, anti-B, anti-Tn, and anti-αGal IgG antibodies up to 3 months from disease onset, we found that the antibody titer remained steady over the time (Figure 5). Asymptomatic patients maintained higher anti-A and anti-B IgG antibody titers over time than symptomatic patients although not significant except for anti-B IgG antibody titer at days 8–12 and days 21–25 follow-up period from disease onset. This gives the confidence that natural antibody titers provide innate immunity against symptomatic disease.

The strength of this analysis is that this is the first study looking into the natural anti-A and anti-B antibody titers from both asymptomatic and symptomatic COVID-19 patients compared to healthy control from both pandemic and pre-pandemic period. The results observed from this analysis may further provide evidence supporting the role of natural antibodies in protecting against symptomatic infection. However, due to the small sample size of the healthy controls from the pandemic period, we could not carry out strong statistical tests to confirm the risk of infection according to the ABH and Lewis phenotypes.

In conclusion, from our observation on natural antibody titers in this cohort, higher natural anti-A and possibly anti-B antibody titers may provide protection against symptomatic infection. These findings could help guide further studies into whether certain blood group and secretor combinations are linked to differences in SARS-CoV-2 susceptibility or severity and potentially support more personalized approaches to understanding immune responses in viral infections.

## Data Availability

The raw data supporting the conclusions of this article will be made available by the authors, without undue reservation.

## Supplementary material

SUPPLEMENTARY FIGURE 1 Comparison of natural antibody titer by age. **(A,E,I)** anti-A antibodies (B and O blood groups) with healthy control <45 years old (*n*=32), healthy control ≥45 years old (*n*=6), asymptomatic patients <45 years old (*n*=13) and ≥45 years old (*n*=2), symptomatic patients <45 years old (*n*=14) and ≥45 years old (*n*=36). **(B,F,J)** Anti-B antibodies (IgG, IgA and IgM) with healthy control <45 years old (*n*=32), healthy control ≥45 years old (*n*=7), asymptomatic patients <45 years old (*n*=11), and ≥45 years old (*n*=5), symptomatic patients <45 years old (*n*=14) and ≥45 years old (*n*=26). **(C,G,K)** Anti-αGal antibodies (IgG, IgA and IgM) with healthy control <45 years old (*n*=49), healthy control ≥45 years old (*n*=9), asymptomatic patients <45 years old (*n*=19), and ≥45 years old (*n*=6), symptomatic patients <45 years old (*n*=25) and ≥45 years old (*n*=49). **(D,H,L)** Anti-Tn mono antibodies (IgG, IgA and IgM) with healthy control <45 years old (*n*=49), healthy control ≥45 years old (*n*=9), asymptomatic patients <45 years old (*n*=19), and ≥45 years old (*n*=6), symptomatic patients <45 years old (*n*=25) and ≥45 years old (*n*=49). *** and ** denotes *p* < 0.005

SUPPLEMENTARY FIGURE 2 Anti-Tn-Mono and Alpha-Gal antibodies among COVID-19 patients and controls. Comparing the natural antibody titers between healthy controls (*n*=59) and COVID-19 patients (*n*=99). **(A)** anti-Tn-Mono IgG antibody titer, **(B)** anti-αGal IgG antibody titer, **(C)** anti-Tn-Mono IgA antibody titer, **(D)** anti-αGal IgA antibody titer **(E)** anti-Tn-Mono IgM antibody titer and **(F)** anti-αGal IgM antibody titer. We stratified antibody titer for anti-Tn-Mono by “non-A” [B+O (*n*=104, patients (65) vs control (39)] and “A” blood group [A+AB (*n*=54, patients (34) vs control (20)] blood groups. We stratified antibody titer for anti-αGal by “non-B” [A+O (*n*=94, patients (55) vs control (39)] and “B” [B+AB (*n*=64, patients (44) vs control (20)] blood groups. Significant difference between patients and controls are presented with black asterisks. Comparisons are also made between blood groups within the healthy controls and patients and only the significant difference is presented with purple asterisks. * *p* = 0.05–0.01, ** denotes *p* = 0.01–0.001 and *** denotes *p* < 0.001.

## References

[1] Akter A Ahmed T Tauheed I Akhtar M Rahman SIA Khaton F. Disease characteristics and serological responses in patients with differing severity of COVID-19 infection: a longitudinal cohort study in Dhaka, Bangladesh. PLoS Negl Trop Dis. (2022) 16:e0010102. doi: 10.1371/journal.pntd.0010102, PMID: 34982773 PMC8759637

[2] Matzhold EM Berghold A Bemelmans MKB Banfi C Stelzl E Kessler HH. Lewis and ABO histo-blood types and the secretor status of patients hospitalized with COVID-19 implicate a role for ABO antibodies in susceptibility to infection with SARS-CoV-2. Transfusion. (2021) 61:2736–45. doi: 10.1111/trf.16567, PMID: 34151460 PMC8447157

[3] Fan Q Zhang W Li B Li DJ Zhang J Zhao F. Association between ABO blood group system and COVID-19 susceptibility in Wuhan. Front Cell Infect Microb. (2020) 10. doi: 10.3389/fcimb.2020.00404, PMID: 32793517 PMC7385064

[4] Göker H Karakulak E Demiroğlu H Ceylan Ç Büyükaşik Y Inkaya A. The effects of blood group types on the risk of COVID-19 infection and its clinical outcome. Turk J Med Sci. (2020) 50:679–83. doi: 10.3906/sag-2005-395, PMID: 32496734 PMC7379446

[5] Kim Y Latz CA DeCarlo CS Lee S Png CYM Kibrik P. Relationship between blood type and outcomes following COVID-19 infection. Semin Vasc Surg. (2021) 34:125–31. doi: 10.1053/j.semvascsurg.2021.05.005, PMID: 34642032 PMC8286549

[6] Velavan TP Pallerla SR Rüter J Augustin Y Kremsner PG Krishna S. Host genetic factors determining COVID-19 susceptibility and severity. EBioMedicine. (2021) 72:103629. doi: 10.1016/j.ebiom.2021.103629, PMID: 34655949 PMC8512556

[7] Liu N Zhang T Ma L Zhang H Wang H Wei W. The impact of ABO blood group on COVID-19 infection risk and mortality: a systematic review and meta-analysis. Blood Rev. (2020) 48:100785. doi: 10.1016/j.blre.2020.100785, PMID: 33309392 PMC7834371

[8] Shamikh Y Salamony A Amer K Elnakib M Hassan W Elzalabany S. Association of blood groups with the clinical presentation of COVID-19 infection. Microb Infect. Dis. (2021) 2:224–231. doi: 10.21608/mid.2021.59111.1111

[9] Wu BB Gu DZ Yu JN Yang J Shen WQ. Association between ABO blood groups and COVID-19 infection, severity and demise: a systematic review and meta-analysis. Infect Genet Evol. (2020) 84:104485. doi: 10.1016/j.meegid.2020.104485, PMID: 32739464 PMC7391292

[10] UI MG-D Hoffmann M Schmidt SL Snyder N Hartmann L. Glycopolymers against pathogen infection. Chem Soc Rev. (2023) 52:2617–42. doi: 10.1039/D2CS00912A, PMID: 36820794

[11] Watanabe Y Bowden TA Wilson IA Crispin M. Exploitation of glycosylation in enveloped virus pathobiology. Biochim Biophys Acta Gen Subj. (2019) 1863:1480–97. doi: 10.1016/j.bbagen.2019.05.012, PMID: 31121217 PMC6686077

[12] Everest-Dass AV Kolarich D Pascovici D Packer NH. Blood group antigen expression is involved in *C. albicans* interaction with buccal epithelial cells. Glycoconj J. (2017) 34:31–50. doi: 10.1007/s10719-016-9726-7, PMID: 27639389

[13] Imbert-Marcille BM Barbé L Dupé M Le Moullac-Vaidye B Besse B Peltier C. A FUT2 gene common polymorphism determines resistance to rotavirus a of the P[8] genotype. J Infect Dis. (2014) 209:1227–30. doi: 10.1093/infdis/jit655, PMID: 24277741

[14] Maroni L van de Graaf SFJ Hohenester SD Oude Elferink RPJ Beuers U. Fucosyltransferase 2: a genetic risk factor for primary Sclerosing cholangitis and Crohn’s disease—a comprehensive review. Clinic Rev Allerg Immunol. (2015) 48:182–91. doi: 10.1007/s12016-014-8423-1, PMID: 24828903

[15] Wipplinger M Mink S Bublitz M Gassner C. Regulation of the Lewis blood group antigen expression: a literature review supplemented with computational analysis. Transfus Med Hemother. (2024) 51:225–36. doi: 10.1159/000538863, PMID: 39135855 PMC11318966

[16] Barbé L Le Moullac-Vaidye B Echasserieau K Bernardeau K Carton T Bovin N. Histo-blood group antigen-binding specificities of human rotaviruses are associated with gastroenteritis but not with in vitro infection. Sci Rep. (2018) 8:12961. doi: 10.1038/s41598-018-31005-4, PMID: 30154494 PMC6113245

[17] Henry S Oriol R Samuelsson B. Lewis Histo-blood group system and associated secretory phenotypes. Vox Sang. (1995) 69:166–82. doi: 10.1111/j.1423-0410.1995.tb02591.x PMID: 8578728

[18] Cheng Y Cheng G Chui CH Lau FY Chan PKS Ng MHL. ABO blood group and susceptibility to severe acute respiratory syndrome. JAMA. (2005) 293:1447–51. doi: 10.1001/jama.293.12.1450-c, PMID: 15784866

[19] Guillon P Clément M Sébille V Rivain JG Chou CF Ruvoën-Clouet N. Inhibition of the interaction between the SARS-CoV spike protein and its cellular receptor by anti-histo-blood group antibodies. Glycobiology. (2008) 18:1085–93. doi: 10.1093/glycob/cwn093, PMID: 18818423 PMC7108609

[20] Breiman A Ruvoën-Clouet N Deleers M Beauvais T Jouand N Rocher J. Low levels of natural anti-α-N-Acetylgalactosamine (Tn) antibodies are associated with COVID-19. Front Microbiol. (2021) 12:12. doi: 10.3389/fmicb.2021.641460, PMID: 33643275 PMC7905038

[21] World Health Organization. Clinical management of COVID-19: Interim guidance, 27 may 2020. Geneva: World Health Organization; (2020). 16, 27–32

[22] Barnkob MB Pottegård A Støvring H Haunstrup TM Homburg K Larsen R. Reduced prevalence of SARS-CoV-2 infection in ABO blood group O. Blood Adv. (2020) 4:4990–3. doi: 10.1182/bloodadvances.2020002657, PMID: 33057631 PMC7594382

[23] Mahmud R Rassel MA Monayem FB Sayeed SKJB Islam MS Islam MM. Association of ABO blood groups with presentation and outcomes of confirmed SARS CoV-2 infection: a prospective study in the largest COVID-19 dedicated hospital in Bangladesh. PLoS One. (2021) 16:e0249252. doi: 10.1371/journal.pone.0249252, PMID: 33826648 PMC8026078

[24] Pendu JL Breiman A Rocher J Dion M Ruvoën-Clouet N. ABO blood types and COVID-19: spurious, anecdotal, or truly important relationships? A reasoned review of available data. Viruses. (2021) 13:160. doi: 10.3390/v13020160, PMID: 33499228 PMC7911989

[25] Franchini M Glingani C Del Fante C Capuzzo M Di Stasi V Rastrelli G. The protective effect of O blood type against SARS-CoV-2 infection. Vox Sang. (2021) 116:249–50. doi: 10.1111/vox.13003, PMID: 32950039 PMC7537255

[26] Wu SC Arthur CM Wang J Verkerke H Josephson CD Kalman D. The SARS-CoV-2 receptor-binding domain preferentially recognizes blood group a. Blood Adv. (2021) 5:1305–9. doi: 10.1182/bloodadvances.2020003259, PMID: 33656534 PMC7929867

[27] Gazi MA Fahim SM Hasan MM Hossaini F Alam MA Hossain MS. Maternal and child FUT2 and FUT3 status demonstrate relationship with gut health, body composition and growth of children in Bangladesh. Sci Rep. (2022) 12:18764. doi: 10.1038/s41598-022-23616-9, PMID: 36335265 PMC9637127

[28] Lee B Dickson DM deCamp AC Ross Colgate E Diehl SA Uddin MI. Histo–blood group antigen phenotype determines susceptibility to genotype-specific rotavirus infections and impacts measures of rotavirus vaccine efficacy. J Infect Dis. (2018) 217:1399–407. doi: 10.1093/infdis/jiy054, PMID: 29390150 PMC5894073

[29] Van Trang N Vu HT Le NT Huang P Jiang X Anh DD. Association between norovirus and rotavirus infection and histo-blood group antigen types in Vietnamese children. J Clin Microbiol. (2014) 52:1366–74. doi: 10.1128/JCM.02927-13, PMID: 24523471 PMC3993640

[30] Mankelow TJ Singleton BK Moura PL Stevens-Hernandez CJ Cogan NM Gyorffy G. Blood group type a secretors are associated with a higher risk of COVID-19 cardiovascular disease complications. eJHaem. (2021) 2:175–87. doi: 10.1002/jha2.180, PMID: 34124710 PMC8176350

[31] Marionneau S Airaud F Bovin NV Pendu JL Ruvoën-Clouet N. Influence of the combined ABO, FUT2 and FUT3 polymorphism on susceptibility to Norwalk virus attachment. J Infect Dis. (2005) 192:1071–7. doi: 10.1086/432546, PMID: 16107962

[32] Payne DC Currier RL Staat MA Sahni LC Selvarangan R Halasa NB. Epidemiologic association between FUT2 secretor status and severe rotavirus gastroenteritis in children in the United States. JAMA Pediatr. (2015) 169:1040–5. doi: 10.1001/jamapediatrics.2015.2002, PMID: 26389824 PMC4856001

[33] Guo M Luo G Lu R Shi W Cheng H Lu Y. Distribution of Lewis and Secretor polymorphisms and corresponding CA19-9 antigen expression in a Chinese population. FEBS Open Bio. (2017) 7:1660–71. doi: 10.1002/2211-5463.12278, PMID: 29123975 PMC5666394

[34] Grönwall C Silverman GJ. Natural IgM: beneficial autoantibodies for the control of inflammatory and autoimmune disease. J Clin Immunol. (2014) 34:12–21. doi: 10.1007/s10875-014-0025-4, PMID: 24691998 PMC4354681

[35] Deleers M Breiman A Daubie V Maggetto C Barreau I Besse T. Covid-19 and blood groups: ABO antibody levels may also matter. Int J Infect Dis. (2021) 104:242–9. doi: 10.1016/j.ijid.2020.12.025, PMID: 33326874 PMC7832075

[36] Tamayo-Velasco Á Peñarrubia-Ponce MJ Álvarez FJ de la Fuente I Pérez-González S Andaluz-Ojeda D. ABO blood system and COVID-19 susceptibility: anti-a and anti-B antibodies are the key points. Front Med. (2022) 9:9. doi: 10.3389/fmed.2022.882477/full PMC9081929 35547235

[37] Wu SC Arthur CM Jan HM Garcia-Beltran WF Patel KR Rathgeber MF. Blood group a enhances SARS-CoV-2 infection. Blood. (2023) 142:742–7. doi: 10.1182/blood.2022018903, PMID: 37367252 PMC10294591

[38] Mikame M Tsuno NH Miura Y Kitazaki H Uchimura D Miyagi T. Anti-a and anti-B titers, age, gender, biochemical parameters, and body mass index in Japanese blood donors. Immunohematology. (2023) 39:155–65. doi: 10.2478/immunohematology-2023-023, PMID: 38179781

