# Supplementary figures and images for: COVID-19 and blood group-related antigens: can natural anti-carbohydrate antibodies provide innate protection from symptomatic SARS-CoV-2 infection?
Source: Front Med (Lausanne). 2025 Apr 30;12:1554785. doi: 10.3389/fmed.2025.1554785 (PMC12074924; doi:10.3389/fmed.2025.1554785)

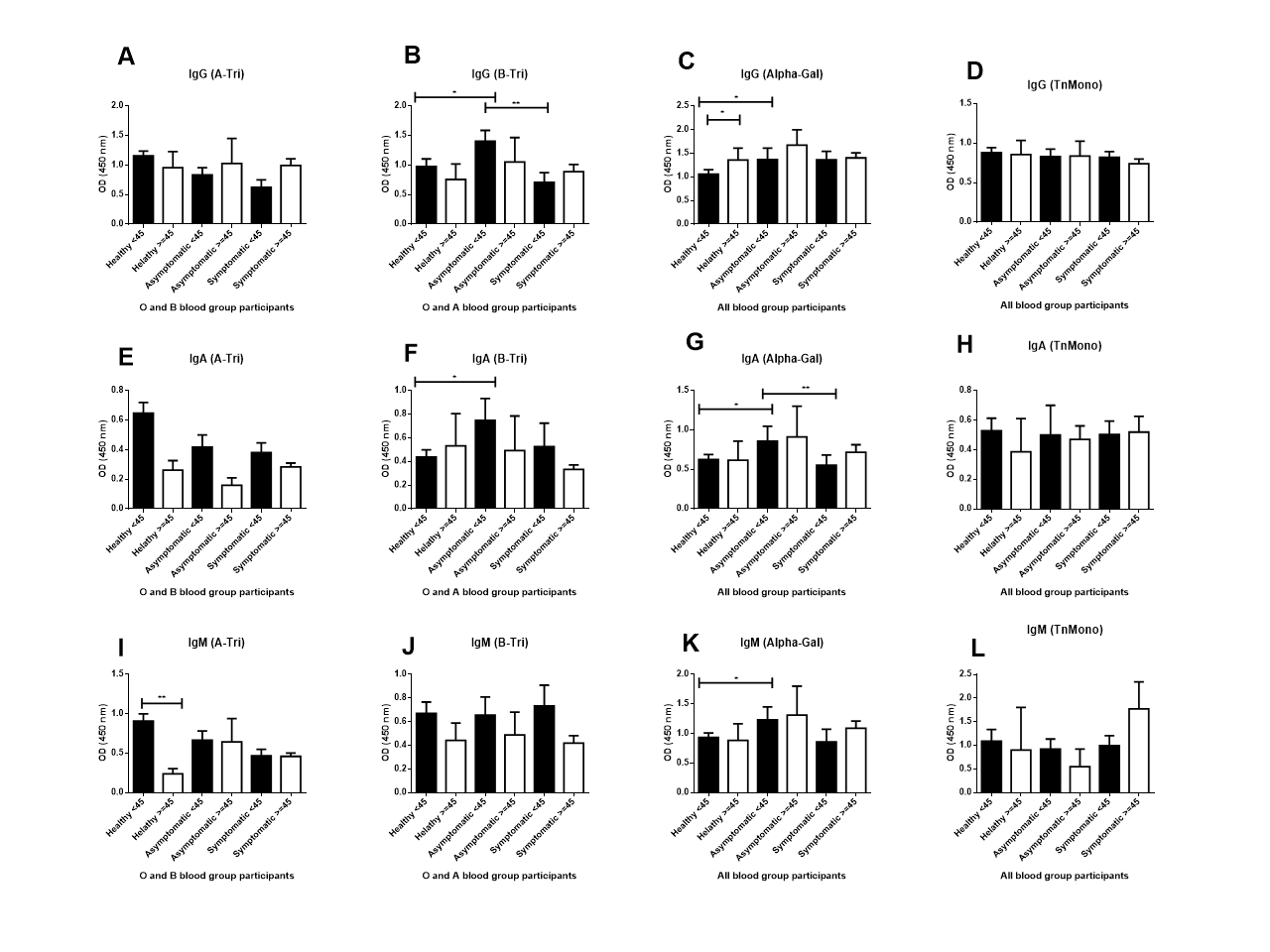

Supplement: SUPPLEMENTARY FIGURE 1 — Comparison of natural antibody titer by age. (A,E,I) anti-A antibodies (B and O blood groups) with healthy control <45 years old (n=32), healthy control ≥45 years old (n=6), asymptomatic patients <45 years old (n=13) and ≥45 years old (n=2), symptomatic patients <45 years old (n=14) and ≥45 years old (n=36). (B,F,J) Anti-B antibodies (IgG, IgA and IgM) with healthy control <45 years old (n=32), healthy control ≥45 years old (n=7), asymptomatic patients <45 years old (n=11), and ≥45 years old (n=5), symptomatic patients <45 years old (n=14) and ≥45 years old (n=26). (C,G,K) Anti-αGal antibodies (IgG, IgA and IgM) with healthy control <45 years old (n=49), healthy control ≥45 years old (n=9), asymptomatic patients <45 years old (n=19), and ≥45 years old (n=6), symptomatic patients <45 years old (n=25) and ≥45 years old (n=49). (D,H,L) Anti-Tn mono antibodies (IgG, IgA and IgM) with healthy control <45 years old (n=49), healthy control ≥45 years old (n=9), asymptomatic patients <45 years old (n=19), and ≥45 years old (n=6), symptomatic patients <45 years old (n=25) and ≥45 years old (n=49). *** and ** denotes p < 0.005 [file Image_1.TIF]

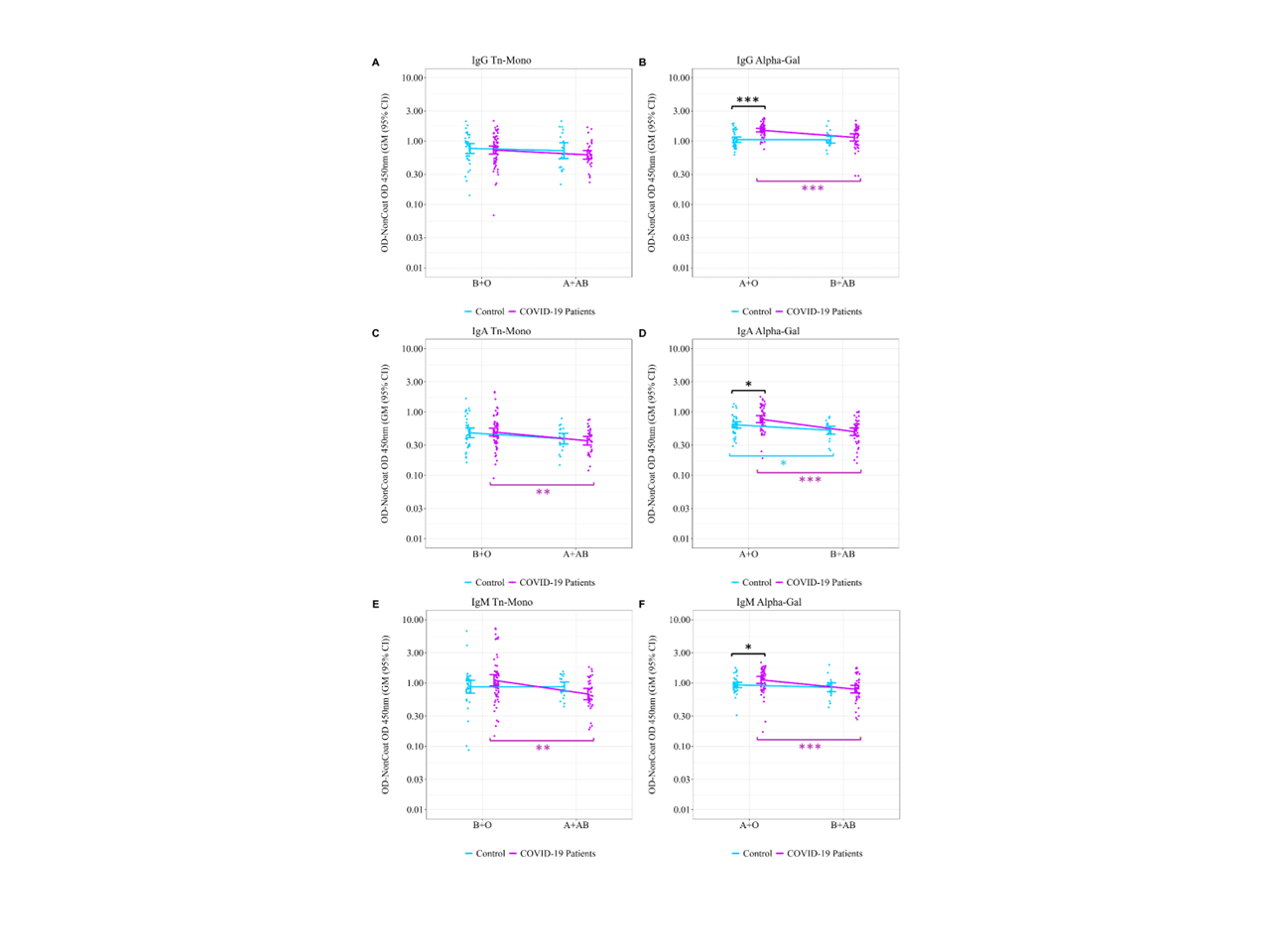

Supplement: SUPPLEMENTARY FIGURE 2 — Anti-Tn-Mono and Alpha-Gal antibodies among COVID-19 patients and controls. Comparing the natural antibody titers between healthy controls (n=59) and COVID-19 patients (n=99). (A) anti-Tn-Mono IgG antibody titer, (B) anti-αGal IgG antibody titer, (C) anti-Tn-Mono IgA antibody titer, (D) anti-αGal IgA antibody titer (E) anti-Tn-Mono IgM antibody titer and (F) anti-αGal IgM antibody titer. We stratified antibody titer for anti-Tn-Mono by “non-A” [B+O (n=104, patients (65) vs control (39)] and “A” blood group [A+AB (n=54, patients (34) vs control (20)] blood groups. We stratified antibody titer for anti-αGal by “non-B” [A+O (n=94, patients (55) vs control (39)] and “B” [B+AB (n=64, patients (44) vs control (20)] blood groups. Significant difference between patients and controls are presented with black asterisks. Comparisons are also made between blood groups within the healthy controls and patients and only the significant difference is presented with purple asterisks. * p = 0.05–0.01, ** denotes p = 0.01–0.001 and *** denotes p < 0.001. [file Image_2.TIF]
