# Supplementary material for: COVID-19 and blood group-related antigens: can natural anti-carbohydrate antibodies provide innate protection from symptomatic SARS-CoV-2 infection?
Source: Front Med (Lausanne). 2025 Apr 30;12:1554785. doi: 10.3389/fmed.2025.1554785 (PMC12074924; doi:10.3389/fmed.2025.1554785)
Supplement: Supplementary file 3 [file Table_1.DOCX]

**Supplementary Table 1 Effect of age and symptoms on the log transformed natural anti-carbohydrate antibody titer**

| **Item** | **Variable** | **IgG** | | **IgA** | | **IgM** | |
| --- | --- | --- | --- | --- | --- | --- | --- |
|  |  | **Coefficient**  **(95% CI)** | **p value** | **Coefficient**  **(95% CI)** | **p value** | **Coefficient**  **(95% CI)** | **p value** |
| Anti-A | Intercept | -0.41 (-2.03, 1.22) | 0.626 | -0.03 (-1.52, 1.45) | 0.964 | 0.02 (-1.40, 1.43) | 0.983 |
|  | Age | 0.00 (-0.04, 0.05) | 0.867 | -0.04 (-0.08, 0.01) | 0.094 | -0.02 (-0.06, 0.02) | 0.374 |
|  | Symptomatic | -1.13 (-2.98, 0.73) | 0.239 | -1.24 (-2.93, 0.46) | 0.158 | -0.77 (-2.39, 0.85) | 0.357 |
|  | Age × Symptomatic | 0.02 (-0.03, 0.07) | 0.504 | 0.04 (-0.01, 0.08) | 0.138 | 0.01 (-0.03, 0.06) | 0.525 |
| Anti-B | Intercept | 0.94 (-0.13, 2.01) | 0.092 | 0.22 (-1.02, 1.46) | 0.732 | -0.43 (-1.81, 0.94) | 0.539 |
|  | Age | -0.02 (-0.05, 0.01) | 0.124 | -0.03 (-0.06, 0.01) | 0.111 | -0.01 (-0.05, 0.03) | 0.578 |
|  | Symptomatic | -2.29 (-3.64, -0.95) | 0.002 | -1.16 (-2.72, 0.39) | 0.148 | 0.72 (-1.00, 2.44) | 0.416 |
|  | Age × Symptomatic | 0.04 (0.01, 0.07) | 0.018 | 0.02 (-0.01, 0.06) | 0.248 | -0.01 (-0.05, 0.03) | 0.490 |
| Anti-Tn | Intercept | -0.19 (-0.92, 0.54) | 0.604 | -0.92 (-1.65, -0.20) | 0.015 | 0.73 (-0.28, 1.75) | 0.158 |
|  | Age | 0.00 (-0.02, 0.02) | 0.761 | 0.00 (-0.02, 0.02) | 0.835 | -0.03 (-0.05, 0.00) | 0.030 |
|  | Symptomatic | 0.07 (-0.81, 0.95) | 0.873 | 0.34 (-0.55, 1.22) | 0.457 | -0.76 (-1.98, 0.47) | 0.230 |
|  | Age × Symptomatic | 0.00 (-0.02, 0.02) | 0.783 | -0.01 (-0.03, 0.01) | 0.524 | 0.03 (0.00, 0.06) | 0.047 |
| Anti-α-gal | Intercept | 0.26 (-0.22, 0.74) | 0.291 | -0.43 (-1.08, 0.22) | 0.197 | 0.23 (-0.40, 0.86) | 0.483 |
|  | Age | 0.00 (-0.01, 0.01) | 0.836 | 0.01 (-0.01, 0.02) | 0.529 | 0.00 (-0.02, 0.01) | 0.831 |
|  | Symptomatic | 0.03 (-0.55, 0.62) | 0.911 | 0.28 (-0.51, 1.06) | 0.494 | 0.17 (-0.59, 0.93) | 0.664 |
|  | Age × Symptomatic | 0.00 (-0.02, 0.01) | 0.811 | -0.01 (-0.03, 0.01) | 0.173 | -0.01 (-0.03, 0.01) | 0.368 |
